# Creativity as a framework for innovation in dental education

**DOI:** 10.3389/froh.2023.1233983

**Published:** 2023-11-03

**Authors:** Samantha J. Byrne, Solange Glasser

**Affiliations:** ^1^ Melbourne Dental School, University of Melbourne, Melbourne, VIC, Australia; ^2^ Melbourne Conservatorium of Music, University of Melbourne, Melbourne, VIC, Australia

**Keywords:** creativity, innovation, dental education, online learning, dental schools

## Abstract

Dental education is rich with examples of innovation as educators have responded to advances in knowledge, technology, the needs of the community, and most recently the challenges of the COVID-19 pandemic. Current challenges requiring innovative pedagogies include developing graduates who are interprofessional collaborative practice-ready, adapting to technological advances, embedding sustainability in the curriculum, and addressing equity and diversity in dental education. Creativity is the production of something that is novel and useful and is intimately linked to innovation which is the implementation of new and improved ways of doing things. To develop innovative pedagogies and address the current challenges facing dental education, educators and dental schools must reflect on the factors necessary for supporting creativity and innovation and seek to remove barriers to or biases against creativity. Here, we discuss the importance of creativity in supporting innovation in dental education, and call for leadership to actively support all elements of creativity for continued innovation to address the challenges we face in educating the future oral health workforce.

## 1. Introduction

Both the practice of dentistry and dental education are rich with innovation. From tooth worms to the ecological plaque hypothesis, from amalgam to adhesive restorative materials, from traditional lectures to problem-based learning and from blackboards and chalk to 3D virtual models: curriculum and pedagogy must constantly adapt to advances in dental knowledge, in technology, in our understanding of how students learn, and the changing oral health needs of the community. Dental educators currently face multiple challenges necessitating innovative solutions. Addressing the global neglect of oral heath requires different models of care, with graduates able to engage in interprofessional collaborative practice and adapt to the diverse needs of the communities in which they will work (1). Graduates must be adaptable to technological advances during their practicing careers such as digital workflows and the impact of learning health systems on dental practice (2, 3). Environmental sustainability must be embedded into dental curriculum to reduce the impact of oral healthcare on the environment (4, 5). And more inclusive, humanistic learning environments are needed to combat equity and diversity in dental education (6).

In this article, we discuss the importance of supporting creativity to continue innovation in dental education. We start by defining creativity and innovation and examine a model of creativity. We then propose creativity as a framework to support innovation. We discuss elements required by individuals and organisations to nurture creativity and innovation, providing examples of these elements from dental education. We consider how these elements relate to a model of creativity and issue a call for action by leadership in dental education to foster these elements in their staff and in their environments, in order to support continued innovation.

## 2. Discussion

### 2.1. Creativity and innovation

Creativity is highly appreciated within organisations, educational settings, and scientific endeavours (7, 8), and is strongly linked to innovation. Creativity is generally viewed as idea generation, and innovation as idea implementation, unsurprisingly leading to a focus on the impact of innovation and creativity as determinants of organisational performance and success (7). Indeed, Anderson and colleagues (2014) proposed the following integrative definition of creativity and innovation:

Creativity and innovation at work are the process, outcomes, and products of attempts to develop and introduce new and improved ways of doing things. The creativity stage of this process refers to idea generation, and innovation refers to the subsequent stage of implementing ideas toward better procedures, practices, or products. Creativity and innovation can occur at the level of the individual, work team, organization, or at more than one of these levels combined but will invariably result in identifiable benefits at one or more of these levels of analysis (7) (p. 1298).

While creativity and innovation are suggested to occur at all levels or combinations of levels of an organisation, creativity has been argued to be primarily an individual process, whereas innovation represents group or social processes (9). Regardless of the potential weight of individual vs. group input into these processes, creativity and innovation remain complex phenomena that require leadership dedicated to fostering and maximising their benefits to ensure improved ways of working (7). Before we discuss how educational institutions can foster creativity and innovation, we will explore definitions and models of creativity, and what these might mean in the context of dental education.

### 2.2. Defining creativity

Creativity can be defined as the production of ideas that are both novel and useful. While this definition speaks to the essence of what creativity is often framed as, it may lack nuance when considering the role of educational systems in fostering creativity. The American Psychological Association (APA) considers creativity to be “The ability to produce or develop original work, theories, techniques, or thoughts. A creative individual typically displays originality, imagination, and expressiveness.” (American Psychological Association, 2018) (10). The APA emphasises what the creative individual would typically display as a result of creative ideation. Perhaps more telling is the assertion that creativity is “The production of ideas and objects that are both novel or original and worthwhile or appropriate, that is, useful, attractive, meaningful, or correct. According to some researchers, in order to qualify as creative, a process of production must in addition be heuristic or open-ended rather than algorithmic (having a definite path to a unique solution) (11).” In this definition, from the Oxford Dictionary of Psychology, we see an amalgamation of two creative elements: the process of production (including the suggestion that this process be open-ended), and the product itself (in this case noted as being either an idea or object). However, there is no mention of the creative person themselves. Evidently, creativity is hard to pin down. We know what it is when we see it, or when we experience it ourselves, but positioning it or even quantifying it is difficult.

Beyond the person, the process, and the product all alluded to in the above definitions, what is missing is an acknowledgement of the environment within which creativity takes places. As we will discuss shortly, this element of environment holds importance when considering how to foster creativity in dental education. Combined, these four elements—the person, process, product, and press (or environment)—form the pillars of the 4 P's model of creativity (12).

#### 2.2.1. A model of creativity

The 4 P's model of creativity has been the most widely adopted creativity framework since the 1960's (12), enabling researchers a structure to scaffold thinking and experimentation concerning creativity. In this model, the creative product is built by the creative person as the result of the creative process, while being supported in a creative environment (*Press*). Despite its widespread adoption, recent reflection on the model has questioned its individualistic vision of creativity. Given that dental education relies on interactive and context-dependent activities and prioritises the performative and relational aspects of the profession (including practitioner-patient interactions), we will take a more contextual and dynamic approach to considering creativity in tertiary education settings by adopting the 5 A's framework of creativity (13). This recent adaptation of the 4 P's model consists of five elements: actor, action, artifact, audience, and affordances.

**Figure 1:**
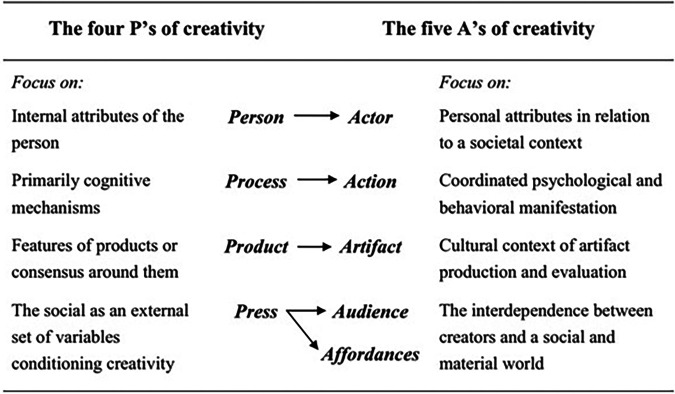
Comparing the four P's and the five A's frameworks (13). [Reprinted from Reviews of General Psychology, 17, Vlad Petre Glăveanu, Rewriting the Language of Creativity: The Five A's Framework, 69–81, Copyright (2012), with permission from SAGE].

A comparison of the two models shows similarities between each of the elements, although the focus of each differs slightly, with the relational or contextual aspect underscored in the 5 A's model (Figure 1). Comparing the models, the final element exhibits the greatest conceptual shift, with *Press*—referring to the “pressing” environmental influences that surround a creative person and their creative product—being divided into two separate categories in Glăveanu's 5A's model: *Audience* and *Affordances*. This division allows us to reflect more deeply on both the social and material environments that a creative dental educator (*Actor*) works with and in. Glăveanu's definition of the audience as “multiple others that assist, contribute, judge, criticise, or use the creative act and/or resulting artifact(s)” (p. 74) is an important distinction that aligns with the role of collaboration, for example through peer-review of teaching for dental educators (14). The *Affordances* of this model speaks to the environment in which educators work, and the role of leadership in fostering creativity in tertiary settings. Learning environments require three key elements to be implemented to ensure creativity is fostered at a tertiary level: designing creative learning environments, facilitating student creativity, and modelling creative pedagogical practice (15). This involves embedding creativity at the level of the learning environment, the student, and the teacher, complementing Beghetto's assertion that creative teaching must include “teaching *about* creativity, teaching *for* creativity, and teaching *with* creativity” (16) (p. 549). We will now discuss the relationship between creativity of individuals and innovation in institutions, giving examples from dental education and relating these examples to the elements of the 5A's model. In doing so, we seek to identify the elements of this framework that appear most relevant for dental education, providing clues as to where leadership can focus their support for creativity and therefore innovation.

### 2.3. Factors facilitating creativity and innovation, and the implications for dental education

**Figure 2:**
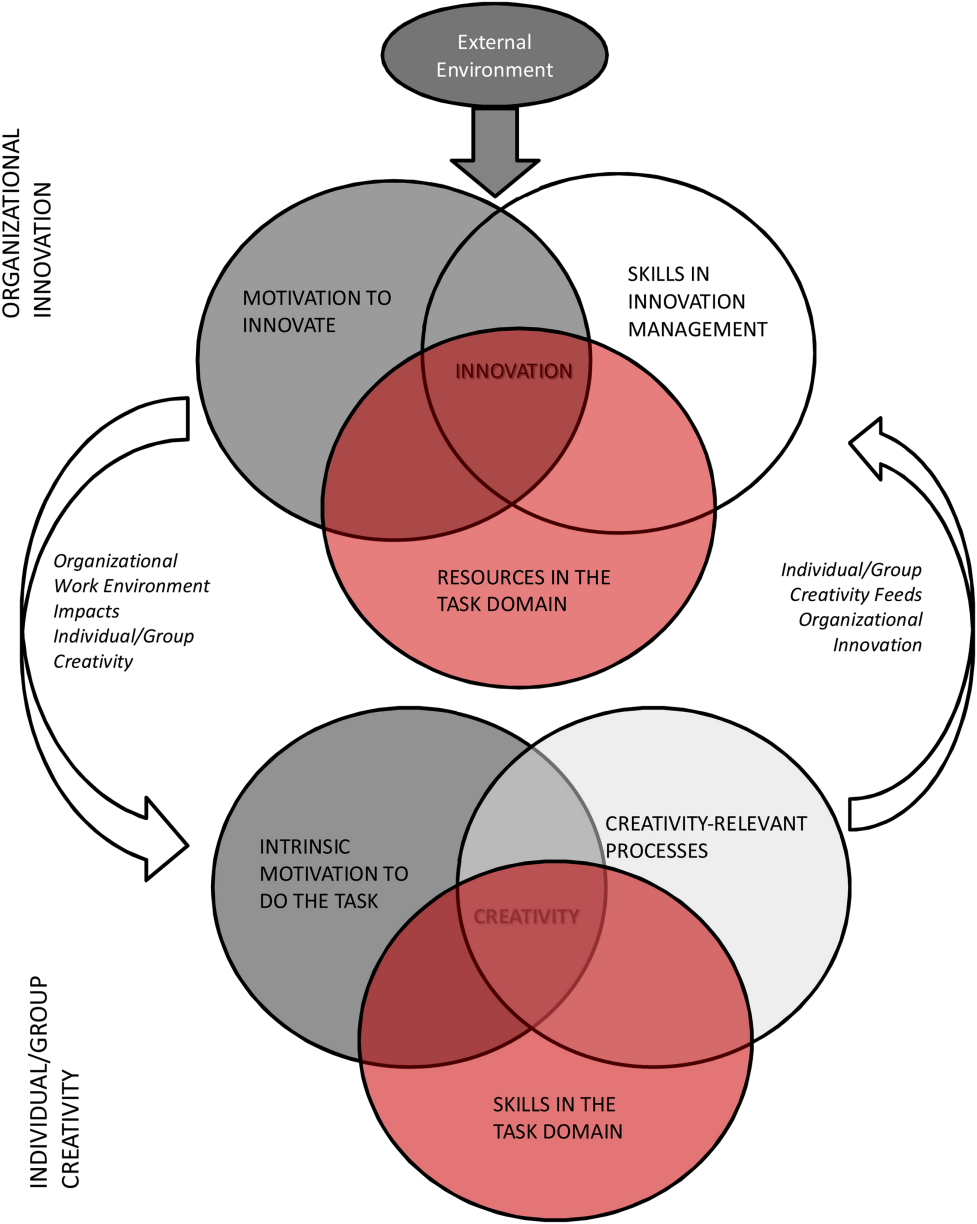
Components influencing innovation and creativity (17). [Reprinted from Research in Organizational Behavior, Vol 36, Amabile TM and Pratt MG, The Dynamic componential model of creativity and innovation in organizations: Making progress, making meaning, 157–183, Copyright (2016), with permission from Elsevier].

As described above, creativity is viewed as the generation of novel and useful ideas, with innovation being the implementation of these. Creativity of individuals and small groups, and innovation in the organisation are closely related (17). The relationship between the two is bidirectional and is based on a correspondence between the factors necessary for individual creativity (see *Actor* and *Action*, Figure 1), and those necessary for institutional innovation (Figure 2). The factors necessary for institutional innovation relate to the environment or the *Audience* and *Affordances* of the 5A's model of creativity (Figure 1).

Individual creativity requires a concurrence of motivation to do the task, expertise in the relevant domain, and creative thinking (17). The COVID-19 pandemic provided the perfect case-study to illustrate the interrelationships between the creativity of dental educators and innovation in educational institutions (18). Educators were motivated to continue teaching and assessing students online enabling them to combine pedagogical and content expertise and creative thinking to develop solutions to students not being able to attend in-person classes (19). These innovations were only successful because educational institutions were open to changing the way students were taught and assessed (20), could manage this innovation, and provided the necessary physical and technological resources.

#### 2.3.1. Expertise in the relevant domain

Our expertise or knowledge is the foundation of our creativity, because new ideas are built on old ideas (21). However, the complexity of problems faced by organisations often necessitates expertise across multiple domains to generate new ideas (17). A useful framework for conceptualising the expertise required of dental educators is the Technological Pedagogical Content Knowledge (TAPCK) framework (22) which illustrates that content, pedagogy and technology must be integrated for effective teaching in the digital age (23).

As dental educators, we come to our roles often because of our content knowledge. However, the challenges facing dental educators may require knowledge across multiple content areas. In their recent discussion of the implications of healthcare challenges towards 2040 on dental education, Reddy and Hughes describe the need to integrate clinical, biomedical, population health and behavioural sciences in dental curricula (24), which requires content knowledge across multiple domains. Such integration of diverse content knowledge can be supported by developing teaching teams from multiple disciplines (25). This has particular relevance for developing interprofessional learning opportunities for oral health professional students. To complement content expertise, knowledge of how students learn is vital for dental educators. In a study of professional development in emerging pedagogies, collaborative development of pedagogical knowledge among dental educators was found to support the implementation of teaching innovation, with a lack of pedagogical knowledge acting as a barrier to adoption of innovative pedagogies (23). Mloka and colleagues reported a similar relationship between pedagogical knowledge and innovation even under challenging circumstances of increasing student numbers, high teaching loads and curriculum change (26). A lack of technological knowledge impedes innovation. Whilst dental educators have been employing a diverse range of technological tools in teaching for decades, barriers to the use of such tools in dental education include the need to understand new and complex technologies and how these can be incorporated into teaching practice (27) and a lack of familiarity with available tools (28). Educators need to understand how to utilise technologies to improve student learning. Employing an innovative self-study methodology, Leadbeatter and colleagues describe the collaborative development of technological knowledge that enabled them to understand technology as dental educators (29). Collectively, this indicates that to support the creativity of dental educators, institutions must encourage staff to work collaboratively to share and build their knowledge across relevant domains of content, pedagogy and technology. The implication for leadership is investment in dental educators as the creative *Actor*.

#### 2.3.2. Creativity-relevant processes

Whilst knowledge is vital for creativity, it can stifle innovation in the absence of creative thinking skills (30). Creative problem solving requires a combination of cognitive processes including problem definition, generation of new ideas, both divergent and convergent thinking (31), thinking broadly, and making unusual associations (18). Methods by which dental educators have demonstrated development of these creativity-relevant cognitive processes include design thinking, scenario planning and establishing professional learning communities. Design thinking is a problem-solving framework which encourages participants to work collaboratively with an open mind and suspension of judgement (32). Wolcott and colleagues recently employed a design thinking approach when leading dental faculty to the development of an innovative dental curriculum (33). Design thinking has also been suggested as an ideal approach for designing dental teaching clinics of the future (3). Scenario planning involves responding to hypothetical “what if” questions to create alternative futures (34). In a recent description of scenario planning in dental education, this method enabled educators to explore new ideas in relation to challenges in dental education including interprofessional collaborative practice, diversity and equity, access to dental care, and advocacy to enhance global oral health outcomes (34). Reviewing the proposed evaluation of scenario planning in dental education, Horvath and Quick describe that through engaging with this activity, educators can develop their creative thinking skills such as generating ideas for other contexts to address the challenges proposed by the scenario planning activity (35).

A professional learning community is a group of people who share and reflect on their practice with the view to grow and learn (36). Reflecting on how this definition relates to the 5A's model of creativity, each member of a professional learning community could be considered both as *Actor* and *Audience*. In a discussion of change management in dental education, Palatta proposes that participation in professional learning communities may enable creative thinking in dental educators (37). Whilst not explicitly identified as a professional learning community, the collaborative self-study approach of Leadbeatter and colleagues demonstrates characteristics of such a community. Exploring their approach (29), creativity-relevant processes are abundantly evident including considering new perspectives on problems and making unusual associations (17). An added benefit of a professional learning community may be a positive impact on the satisfaction and morale of dental educators (37). Therefore, returning to the 5A's model of creativity, design thinking, scenario planning, and the development of professional learning communities are collaborative *Affordances* which may enable the development of creativity-relevant attributes of dental educators who each play a dual role as *Actor* and *Audience*.

#### 2.3.3. Motivation

Motivation, particularly intrinsic motivation, is vital for creativity (38). Self-determination theory proposes that satisfying the three basic psychological needs for competence, autonomy and relatedness will enhance motivation (39). This suggests that educators need to believe they have the requisite knowledge and skills, have choice in how they enact their role, and a sense of belonging to a community with similar goals (38) in order to develop innovative solutions to the challenges facing dental education into the future. This aligns with the importance of developing pedagogical knowledge as illustrated by the TPACK framework discussed above. To see this in action, as we saw earlier, in a study of the impact of a professional development program to support the development of pedagogical knowledge, followed by formation of a learning community, dental educators reported they were motivated to improve their teaching practice, with reports of implementation of innovative teaching strategies (23). In a recent systematic review of the use of digital technologies in dental education, Zitzmann and colleagues assert that a high level of motivation is needed for educators to embrace an implement innovative digital technologies (40) and speculate that the digital infrastructure and the level of innovation of educators will be included in the raking of dental schools (40). Therefore, this combination of intrinsic and extrinsic motivation relates to both the *Actor* and *Affordances* of the 5A's model of creativity.

#### 2.3.4. Institutional factors

To this point, we have focused on factors that can enhance the creativity of individuals and teams of educators, and provided examples of how this can influence innovation in dental education. To support creativity and innovation we also need to look to contextual factors in organisations (7), which are conceptualised by Amabile and Pratt (2016) as motivation to innovate, relevant resources, and skills in innovation management (Figure 2) (17). These factors clearly align with *Affordance* in the 5A's model of creativity. Resources include financing for projects, infrastructure with the necessary materials and services, and enabling sufficient time to explore and implement creative solutions (17). For example, MacNeill and Hilario suggest that clinical placement operations that do not enable students from different health professions to interact and engage in integrated patient care is inhibiting interprofessional education (IPE) across dental schools in the US. They further suggest that dental schools need to explore external, community-based models of care to enable authentic interprofessional learning experiences (41). Such a solution requires resourcing in the form of time, financing and developing the appropriate model of care. A successful example of this can be found in a community-based IPE program that enabled nursing and dental students to engage in a collaborative care program, and successfully increased the oral health knowledge of program participants (42). Lack of various resources have been reported as barriers to implementing online learning in dental education. Over a decade has passed since Shonwetter and colleagues reported the greatest impediments to innovation in online learning in dentistry were institutional, including financial cost, technical support required, politics and a stakeholder resistance to change (27), highlighting the need for institutional support for innovation. Time or lack thereof is repeatedly reported as a barrier to implementing innovative pedagogies (28, 37, 43). Institutions must value the time it takes to develop and implement innovative approaches to dental education.

## 3. Conclusion

Through the examples outlined in this article, we demonstrate that some dental educators are leading the way in curriculum innovation, and that this is related to various elements of creativity. However, to continue developing innovative pedagogical practices and to address future challenges in dental education, more action is needed. Just as ‘*Action*’ refers to coordinated psychological and behavioral manifestations in the model of creativity used to scaffold this discussion, we call on leadership in dental education to coordinate action to enable environments which foster and value creativity.

## Data Availability

The original contributions presented in the study are included in the article/Supplementary Material, further inquiries can be directed to the corresponding author.
